# Supplementary material for: Developmental dynamics of catshark cranial neural crest cells provide insights into gnathostome facial evolution
Source: Development. 2026 May 7;153(9):dev205258. doi: 10.1242/dev.205258 (PMC13200729; doi:10.1242/dev.205258)
Supplement: Supplementary information [file develop-153-205258-s1.pdf]

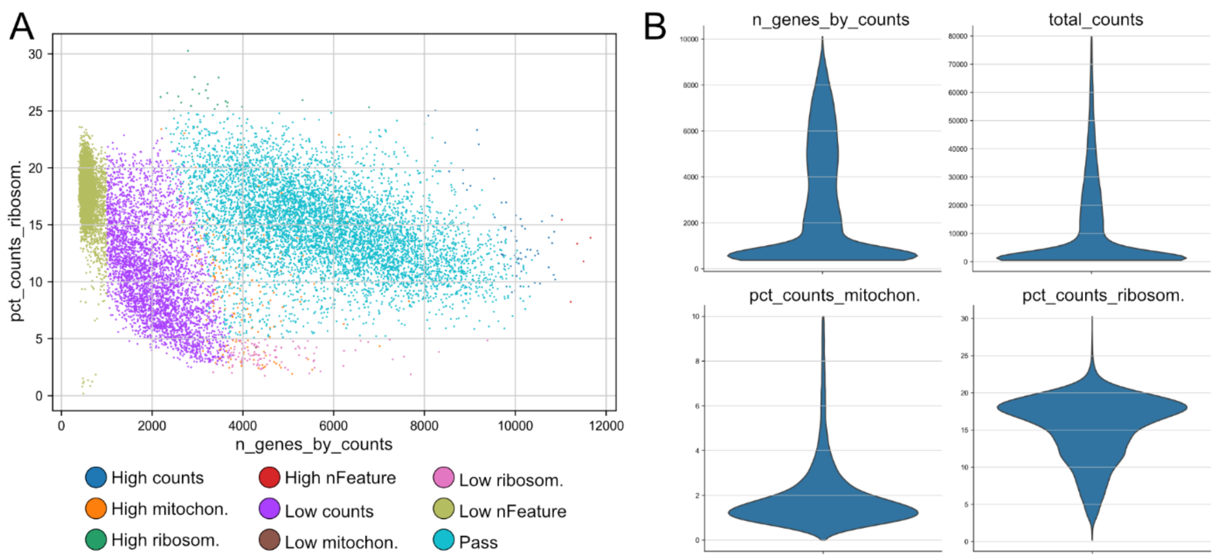

Fig. S1. Quality control overview of the scRNA-seq dataset. (A) Scatter plot highlighting the cells that passed the quality control (QC) check. Passing cells are coloured in light blue. The x-axis shows the number of unique genes found per cell, and the y-axis shows the percentage of ribosomal counts per cell. Cells are coloured based on the reason behind failing the QC. Cells that failed multiple QC categories are coloured based on the first failed category. Doublets were predicted using Scrublet (https://github.com/swolock/scrublet). Cutoff values for each category are provided in the Methods section. **(B)** Violin plots showing the number of genes detected with at least one count per cell (n_genes_by_counts), total number of transcripts detected per cell (total_counts), percentage of mitochondrial counts per cell (pct_counts_mitochon.), and percentage of ribosomal counts per cell (pct_counts_ribosom.).

Development • Supplementary information

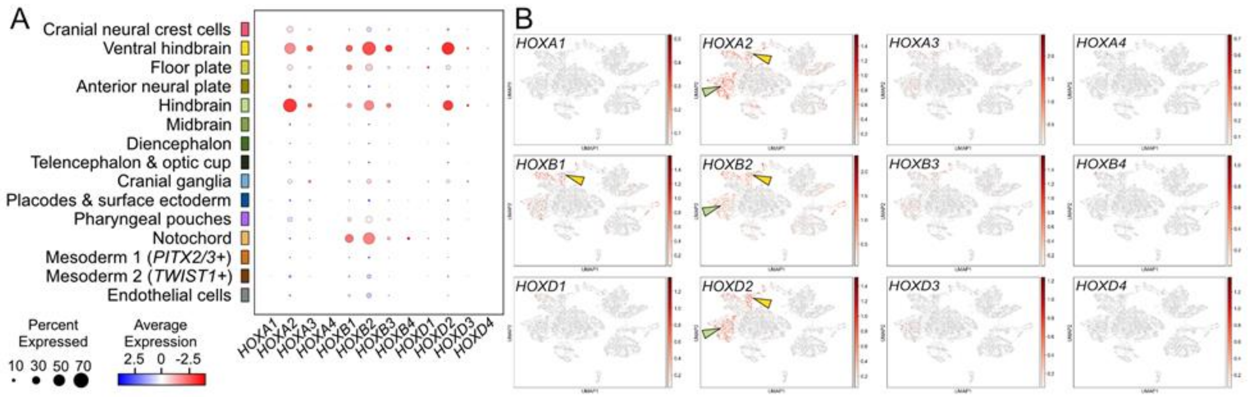

Fig. S2. Screening for hyoid and branchial CNCC. Dotplot **(A)** and feature plots **(B)** of *HOX* genes expressed in hyoid and branchial CNCCs in the catshark based on (Oulion et al., 2011). There is strong *HOXA2*, *HOXB2*, and *HOXD2* expression within the hindbrain and ventral hindbrain clusters that likely corresponds to the anterior part of this brain region (rhombomeres 2-4). There is also a small subset of cells within the same two hindbrain clusters that express high levels of *HOXA3* and *HOXB3*, which likely belong to a more posterior region of the hindbrain (rhombomeres 4-6). The small subset of *HOXB1+* cells corresponds to rhombomere 4. We observe no *HOXA4*, *HOXB4*, and *HOXD4* expression since they are restricted to the posterior-most region of the developing head, which was not included in the single-cell experiment. Note that there is no *HOX* expression within the CNCC cluster, indicating that it is entirely composed of trigeminal CNCC.

Development • Supplementary information

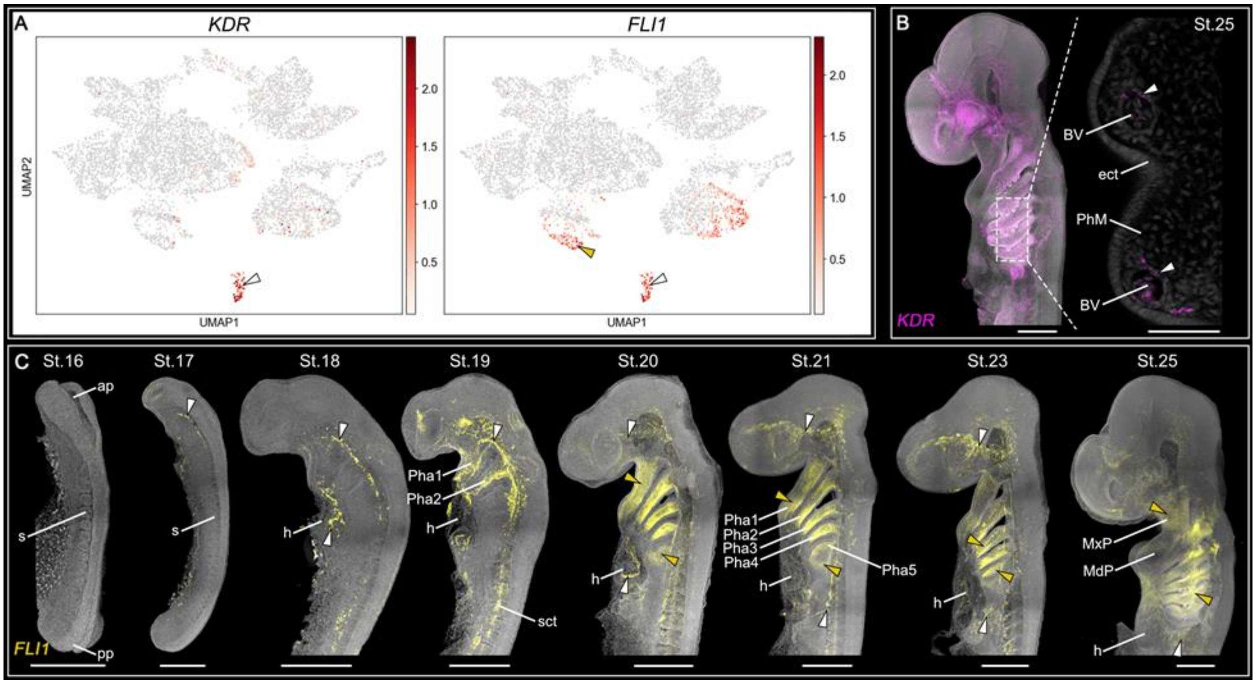

Fig. S3. Endothelial cells scRNA-seq cell cluster validations. (A) Feature plots of *FLI1* and *KDR*. White arrowheads indicate expression in the blood vessels, and yellow arrowheads indicate expression in the facial ectomesenchyme. **(B)** *KDR* expression pattern at St.25. Whole-mount image shows *KDR* expression along the entire circulatory system (left). Scale bar: 500µm. Optical cross-section shows *KDR* expression in the endothelial cells (right). Scale bar: 100µm. **(C)** *FLI1* expression pattern along small-spotted catshark embryogenesis. Arrowheads are coloured following the consensus, as shown in (A). Expression in the mesoderm is covered by the pharyngeal arches, ectomesenchyme, and developing blood vessels. Scale bars: 500µm. ap, anterior neuropore; BV, blood vessel; ect, ectoderm; h, heart; Pha1-5, first to fifth pharyngeal arches; MdP, mandibular prominence; MxP, maxillary prominence; PhM, pharyngeal ectomesenchyme; s, somite.

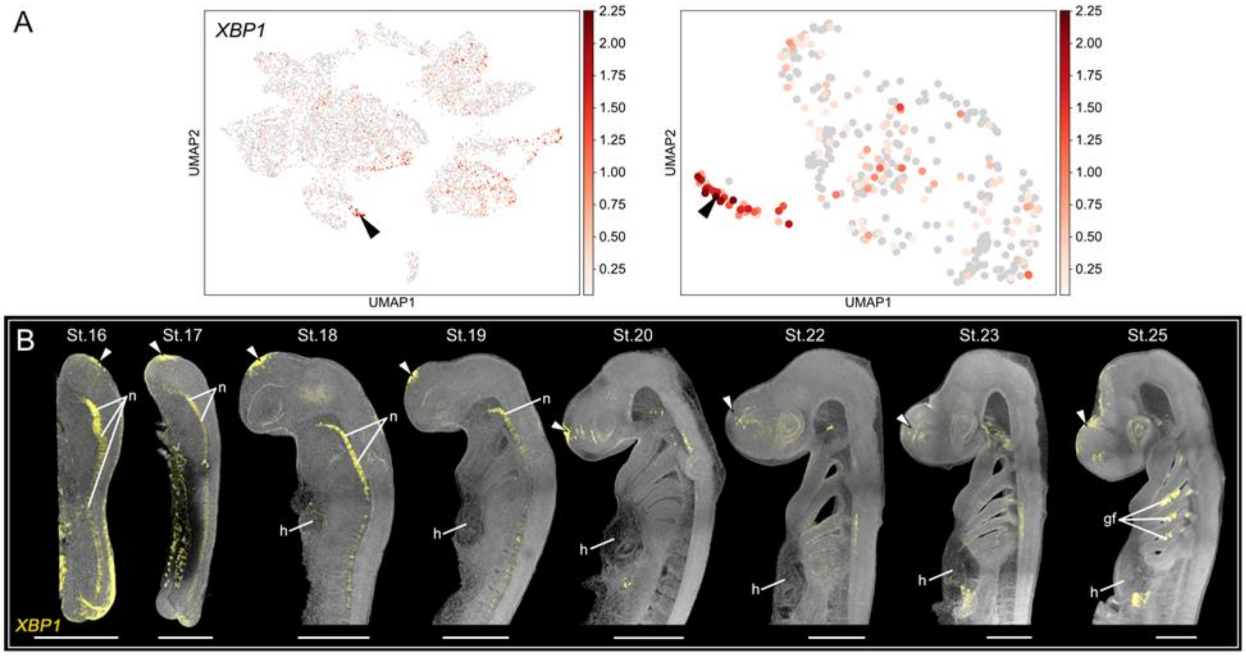

Fig. S4. The small-spotted catshark hatching gland. **(A)** Feature plots of the hatching gland marker gene *XBP1*. Whole head scRNA-seq dataset (left) and CNCC subset (right). **(B)** *XBP1* expression pattern along small-spotted catshark embryogenesis. White arrowheads indicate the expression of *XBP1* in the developing hatching gland. Scale bars: 500µm. gf, gill filaments; h, heart; n, notochord.

Development • Supplementary information

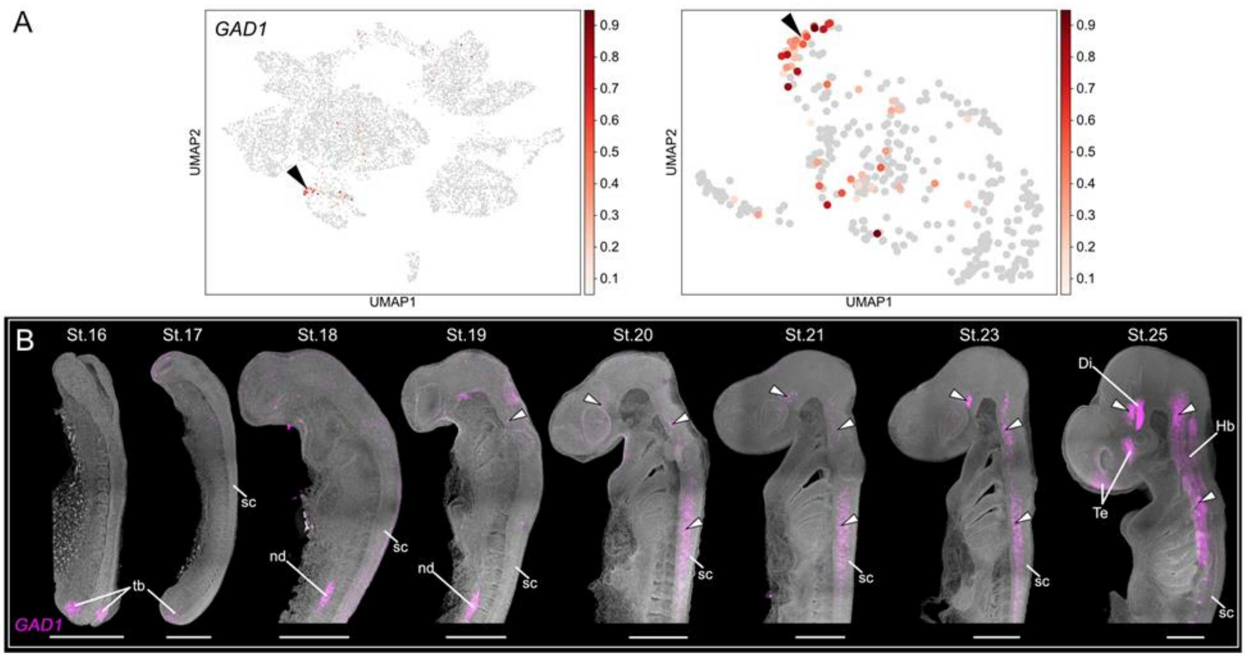

Fig. S5. The small-spotted catshark *GAD1*+ neurons. (A) Feature plots of *GAD1*. Whole head scRNA-seq dataset (left) and CNCC subset (right). **(B)** *GAD1* expression pattern along small-spotted catshark embryogenesis. White arrowheads indicate the expression of *GAD1* in neurons of the developing central nervous system. Scale bars: 500µm. Di, diencephalon; Hb, hindbrain; nd, nephric duct; sc, spinal cord; tb, tailbud; Te, telencephalon.

Development • Supplementary information

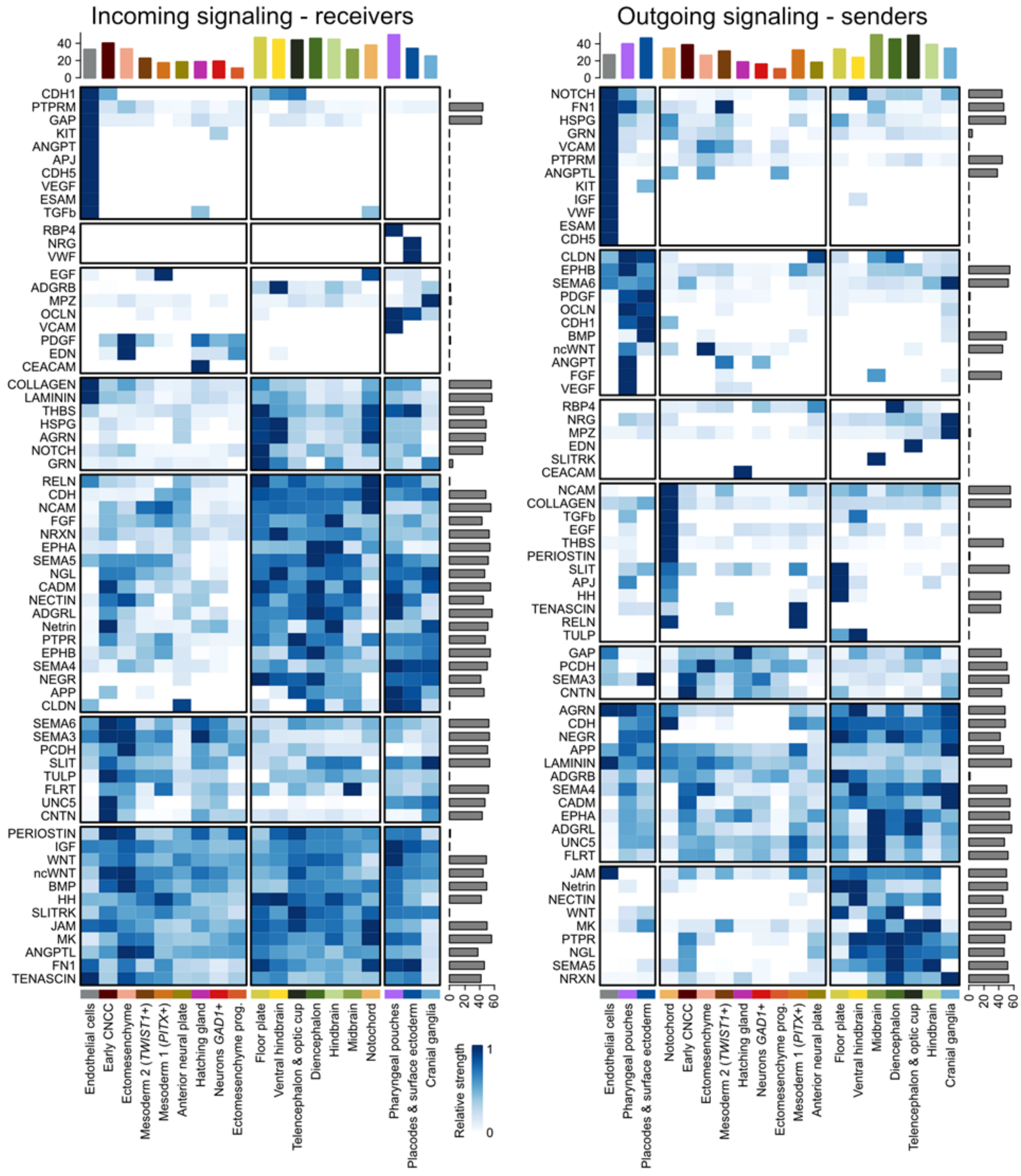

Fig. S6. Raw CellChat cell-cell communication predictions. Heatmaps display the relative signaling strength by cell cluster and per pathway, divided by incoming (left) and outgoing (right) signaling. Columns represent the 19 cell clusters of the scRNA-seq dataset (the five CNCC subclusteres were included in the analysis separately). Rows represent the different signaling pathways. The relative signaling strength per pathway is represented by the blue colors. Cell clusters and signaling pathways were grouped based on similar signaling patterns. Coloured bars on top of the heatmaps indicate the contribution of each cell cluster´s signaling strength to the total predicted signaling strength of the whole dataset. Gray bars on the right of the heatmaps represent the signaling strength of each pathway compared to the total predicted signaling.

Development • Supplementary information

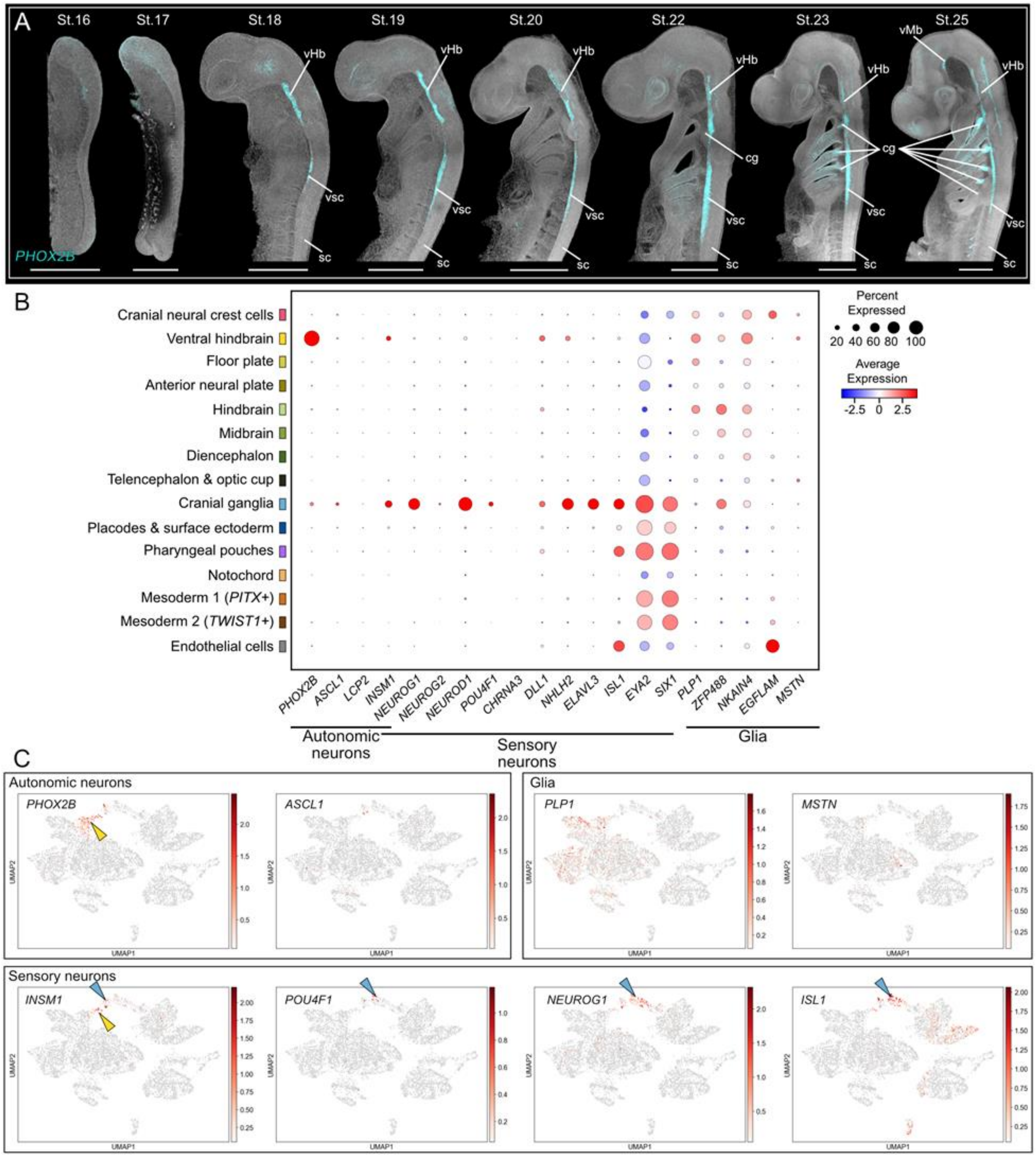

Fig. S7. Screening for CNCC-derived neuroglia. (A) *PHOX2B* expression pattern along small-spotted catshark embryogenesis. Scale bars: 500µm. **(B)** Dotplot of neuroglia marker genes based on (Soldatov et al., 2019). Note that most of the genes are either lowly expressed or absent from the dataset. **(C)** Feature plots of selected neuroglia marker genes from (B). cg, cranial ganglia; sc, spinal cord; vHb, ventral hindbrain; vMb, ventral midbrain; vsc, ventral spinal cord.

Development • Supplementary information

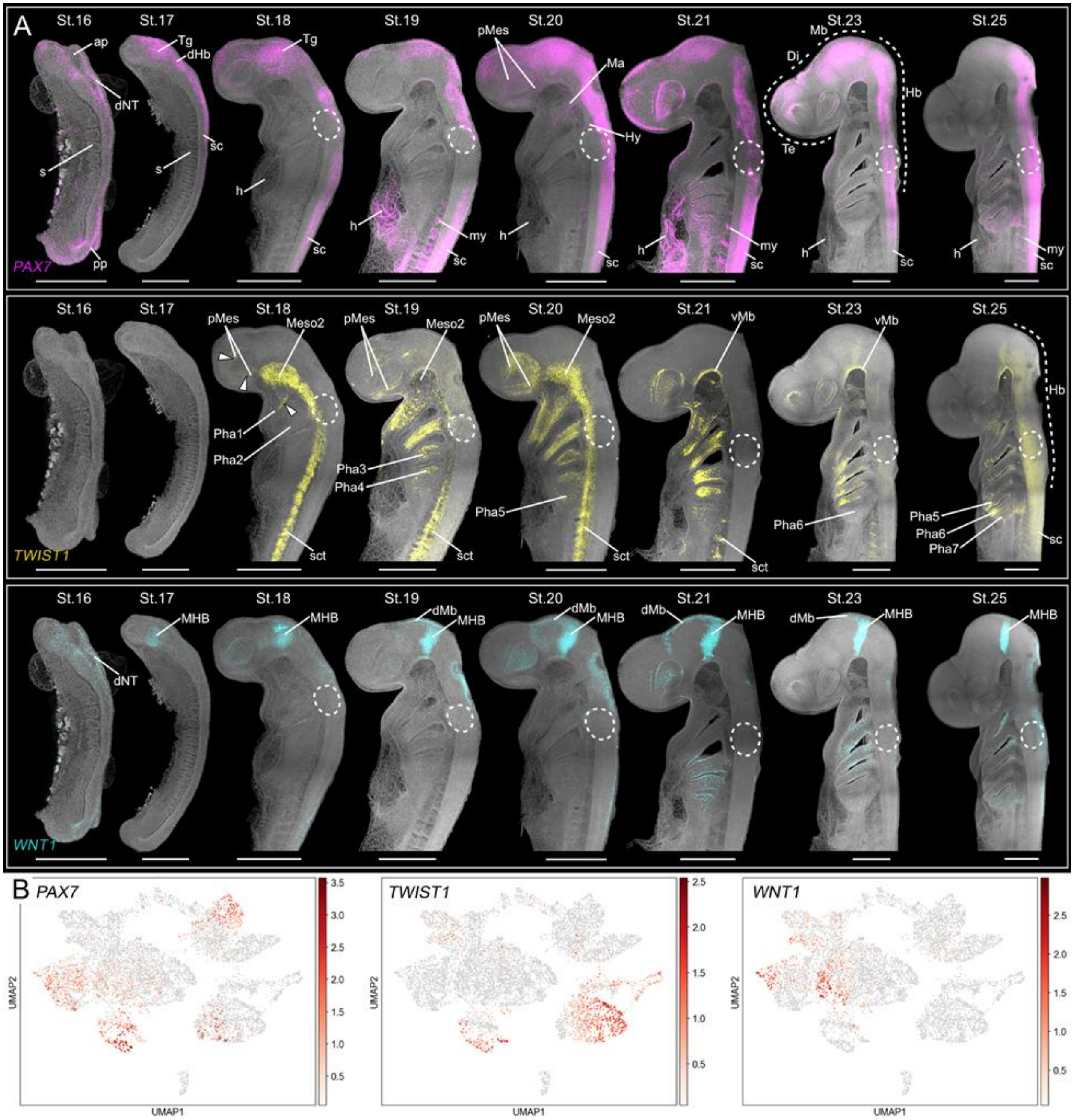

Fig. S8. Split-channel images of the HCR validations for facial ectomesenchyme. (A) 3D confocal laser microscopy split-channel images from Fig. 5A. *PAX7* (magenta), *TWIST1* (yellow), and *WNT1* (cyan). White arrowheads in the *TWIST1* panel indicate the first signs of *TWIST1* expression in the facial ectomesenchyme at St.18 (both, periocular and pharyngeal). White dashed circles indicate the position of the otic capsule. Scale bars: 500µm. **(B)** Feature plots of *PAX7*, *TWIST1*, and *WNT1*. an, anterior neuropore; dHb, dorsal hindbrain; Di, diencephalon; dMb, dorsal midbrain; dNT, dorsal neural tube; h, heart; Hb, hindbrain; Hy, hyoid stream; Ma, mandibular branch; Mb, midbrain; Meso2, mesoderm 2; MHB, midbrain hindbrain boundary; my, myotome; pMes, periocular ectomesenchyme; Pha1-7, first to seventh pharyngeal arches; pp, posterior neuropore; Te, telencephalon; Tg, trigeminal stream; s, somite; sc, spinal cord; sct, sclerotome; vMb, ventral midbrain.

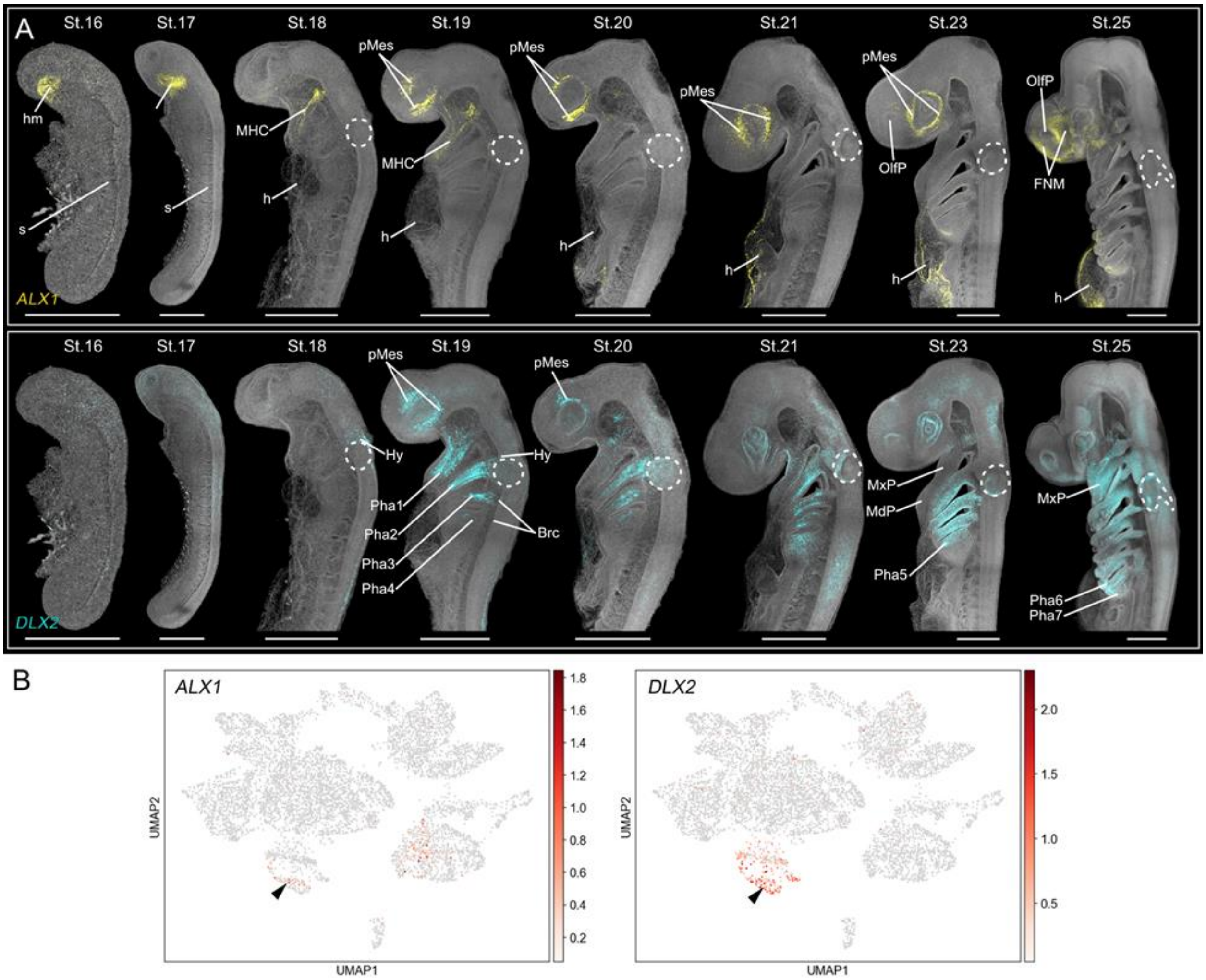

Fig. S9. Split-channel images of the HCR validations for facial ectomesenchyme. (A) 3D confocal laser microscopy split-channel images from Fig. 5B. *ALX1* (yellow) and *DLX2* (cyan). *DLX2* expression can be observed in the periocular region together with *ALX1* from St.19-20. White dashed circles indicate the position of the otic capsule. Scale bars: 500µm. **(B)** Feature plots of *ALX1* and *DLX2*. Brc, branchial CNCC; FNM, frontonasal ectomesenchyme; h, heart; hm, head mesoderm; Hy, hyoid CNCC; MdP, mandibular prominence; MHC, mandibular head cavity; MxP, maxillary prominence; Pha1-7, first to seventh pharyngeal arches; pMes, periocular ectomesenchyme; OlfP, olfactory placode; s, somites.

Development • Supplementary information

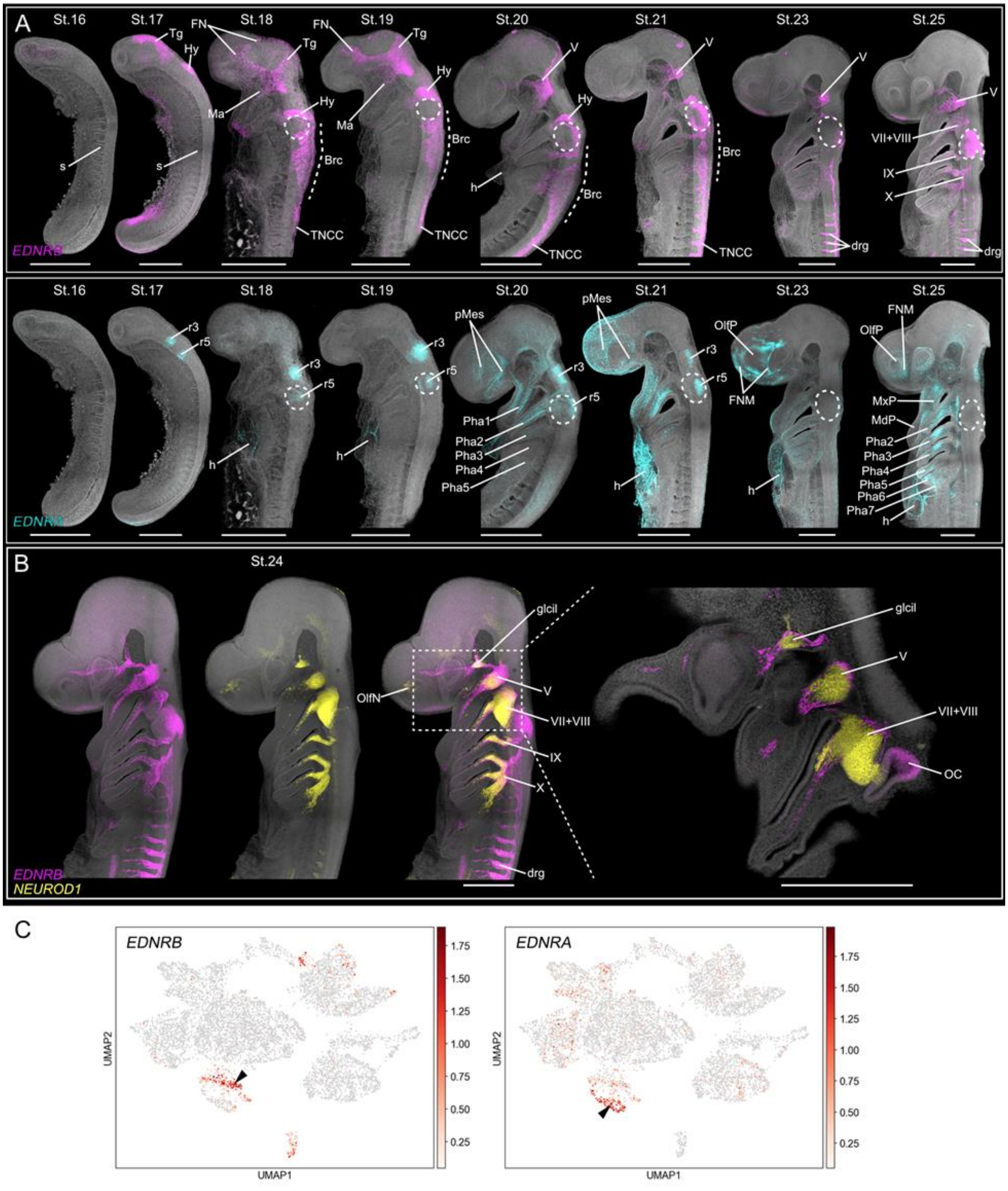

Fig. S10. Split-channel images of the HCR validations for facial ectomesenchyme. (A) 3D confocal laser microscopy split-channel images from Fig. 5C. *EDNRB* (magenta) and *EDNRA* (cyan). Scale bars: 500µm. **(B)** 3D confocal laser microscopy (left) and virtual section (right) of *EDNRB* (magenta) and *NEUROD1* (yellow). Both genes label distinct cellular populations within

Development • Supplementary information

the peripheral nervus system. **(C)** Feature plots of *EDNRB* and *EDNRA*. Brc, branchial CNCC; drg, dorsal root ganglia; FN, frontonasal branch; FNM, frontonasal ectomesenchyme; glcil, ciliary ganglion; h, heart; Hy, hyoid CNCC; IX, glossopharyngeal ganglia; Ma, mandibular branch; MdP, mandibular prominence; MxP, maxillary prominence; OC, otic capsule; OlfP, olfactory placode; Pha1-7, first to seventh pharyngeal arches; pMes, periocular ectomesenchyme; r3-5, third and fifth rhombomeres; s, somites; Tg, trigeminal stream; TNCC, trunk NCC; V, trigeminal ganglia; VII+VIII, acousticofacial ganglia; IX, glossopharyngeal ganglia; X, vagus ganglia.

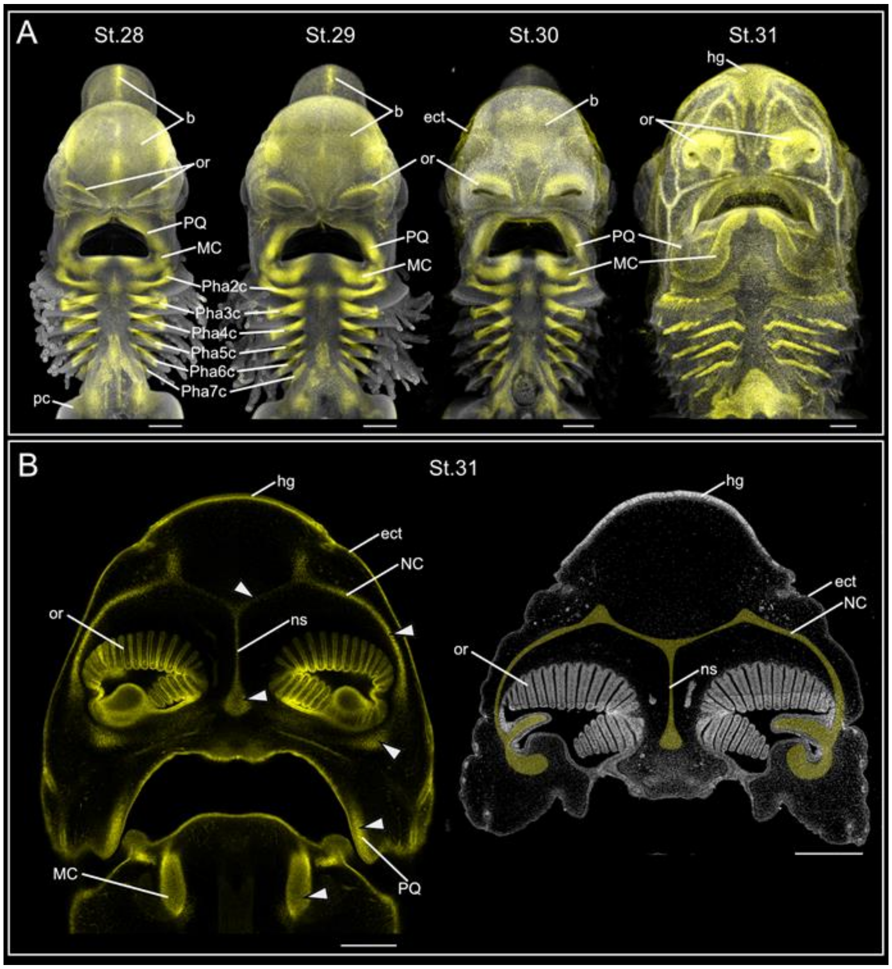

Fig. S11. Immunofluorescence validations of cartilage and chondrogenic mesenchymal condensations SRµCT modelling. (A) Whole-mount immunofluorescence against the SOX9 protein by confocal laser microscopy. Scale bars: 500µm. **(B)** Frontal optical section of the St.31 embryo (left) and SRµCT tomographic slice highlighting the mesenchymal condensation of the nasal capsule. White arrowheads indicate the mesenchymal condensations and cartilaginous elements labelled by the SOX9 antibody. Note the almost identical shape of the nasal capsule in both images. Scale bars: 500µm. b, brain; ect, ectoderm; hg, hatching gland; MC, Meckel's cartilage; NC, nasal capsule; ns, nasal septum; or, olfactory rosette; Pha2c-7c, cartilage/mesenchymal condensations of the second to seventh pharyngeal arches; PQ, palatoquadrate.

Development • Supplementary information

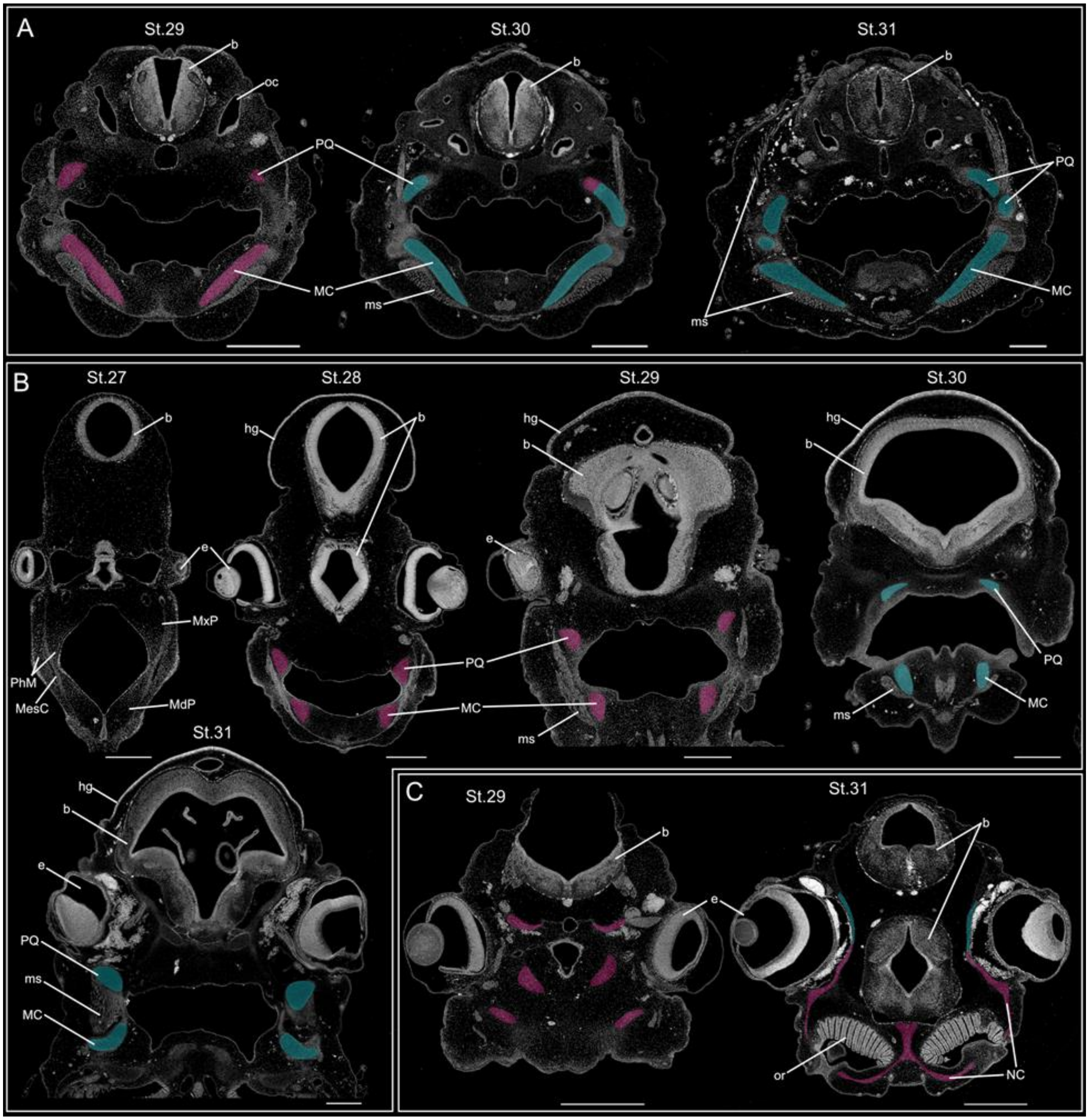

Fig. S12. SRµCT tomographic slices highlighting the segmented chondrogenic elements. (A) Coronal tomographic slices highlighting the palatoquadrate and Meckel's cartilage. Mesenchymal condensations are labelled in magenta, and cartilage in cyan. Scale bars: 500µm. **(B)** Frontal tomographic slices highlighting the palatoquadrate and Meckel's cartilage. Note that there are no highlighted regions in the St.27 tomographic slice since there are no condensations at this stage. Scale bars: 500µm. **(C)** Coronal tomographic slices at the level of the eyes showing the location of the different mesenchymal condensations. Scale bars: 500µm. b, brain; e, eye; hg, hatching gland; MC, Meckel's cartilage; MdP, mandibular prominence; MesC, mesodermal core of pharyngeal arch; ms, muscle; MxP, maxillary prominence; NC, nasal capsule; oc, otic capsule; or, olfactory rosette; PhM, pharyngeal arch ectomesenchyme; PQ, palatoquadrate.

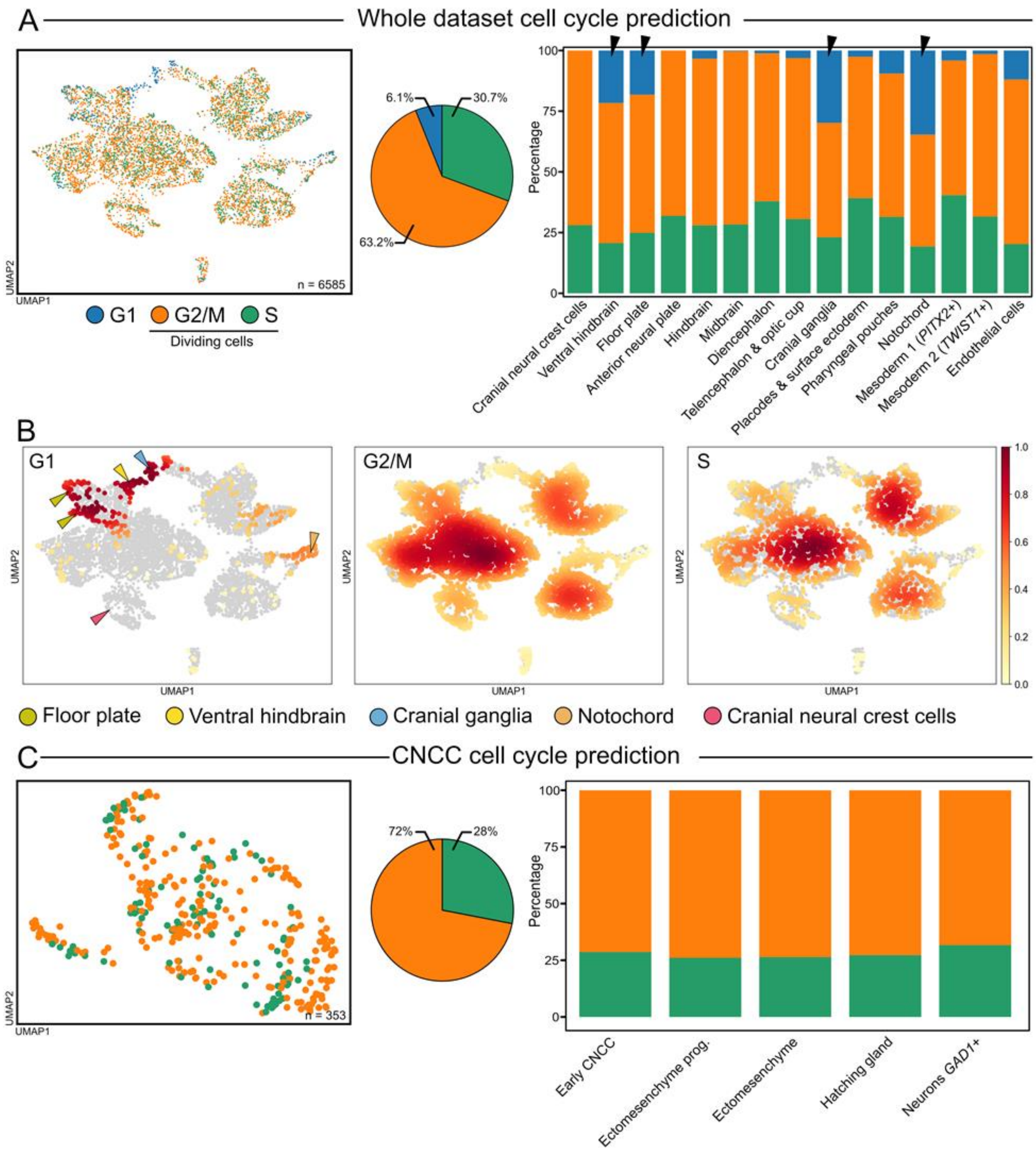

Fig. S13. Cell cycle predictions of the scRNA-seq dataset. (A) Whole head scRNA-seq dataset cell cycle predictions. Distribution of cell cycle phases visualized in the UMAP (left). Pie chart showing the proportions of cells within each cell cycle phase of the whole dataset (middle). Barplot indicating the proportion of cells within each cell cycle phase per cell cluster (right). Black arrowheads indicate the cell clusters with the highest proportion of cells in G1 phase. **(B)** Density of cells per cell cycle phase. Note that cells in G2/M and S phase are evenly distributed across the UMAP. However, cells in the G1 phase are concentrated within certain locations. Arrowheads are

Development • Supplementary information

coloured based on the cell clusters from Fig. 1B and indicate the cell cluster with the highest proportion of G1 cells. **(C)** Cell cycle predictions for the CNCC subset. Distribution of cell cycle phases visualized in the CNCC subset UMAP (left). Pie chart showing the proportions of cells within each cell cycle phase of the whole CNCC subset (middle). Note that there are no cells in G1 phase. Barplot showing the proportion of cells within each cell cycle phase per cell cluster (right). Note that the proportions of cells in G2/M and S phase are consistent throughout the five cell clusters.

Development: doi:10.1242/dev.205258: Supplementary information

Table S1. Cellular sources of *POSTN* during embryogenesis across representative gnathostome species. *POSTN*-expressing cell types and tissues according to single-cell transcriptomic profiles from developing mouse (*Mus musculus*), chicken (*Gallus gallus*), Western clawed frog (*Xenopus tropicalis*), and zebrafish (*Danio rerio*). Since the information was acquired from online interactive resources, normalization between datasets was not performed. Zebrafish possess two *postn* paralogs (*postna* and *postnb*), however, only *postnb* expression was observed in the single-cell datasets.

Species	Dataset	Embryonic stage	Cell types and tissues
Mouse	(Ibarra-Soria et al., 2018)	Whole embryo E8.25	Amnion
	(Pijuan-Sala et al., 2019)	Whole embryo E6.5-8.5	Mesenchyme<br>Allantois
	(Qiu et al., 2024)	Whole-embryo E8.5-9.75	Lateral plate mesoderm<br>Intermediate mesoderm<br>Somites<br>Neural crest (PNS glia)<br>Epithelial cells<br>Gut
Chicken	(Rito et al., 2025)	Trunk region HH8-11	Notochord
Western clawed frog	(Briggs et al., 2018)	Whole embryo St12-22	Notochord
Zebrafish	*postna* (Lange et al., 2024)	Whole embryo 16hpf-2dpf	-
	postnb (Lange et al., 2024)	Whole embryo 16hpf	Somite<br>Periderm
		Whole embryo 19hpf	Myotome<br>Epidermal cells<br>Otic placode<br>Pharyngeal arch
		Whole embryo 1dpf	Blood vasculature<br>Fin<br>Endoderm<br>Somite<br>Epidermal cells<br>Pharyngeal arches 3-7
		Whole embryo 2dpf	Epithelial cell<br>Median fin fold<br>Musculature system<br>Pectoral fin<br>Somite<br>Pectoral fin cartilage<br>Gut<br>Pharyngeal arch
	postna (Wagner et al., 2018)	Whole embryo 4hpf-24hpf	-
	postnb (Wagner et al., 2018)	Whole embryo 4hpf-24hpf	Periderm<br>Endothelium<br>Pharyngeal arch mesoderm<br>Heart<br>Pectoral fin bud

Available for download at
https://journals.biologists.com/dev/article-lookup/doi/10.1242/dev.205258#supplementary-data

Table S2. Small-spotted catshark orthologs to mouse cell cycle genes. List of small-spotted catshark cell cycle orthologs. The list of mouse cell cycle genes was obtained from Tirosh *et al*. (Tirosh et al., 2016). Out of the 97 mouse cell cycle genes, 93 orthologs were successfully identified in the small-spotted catshark. The 4 orthologs that were not detected are labelled with a hyphen (-).

Available for download at
https://journals.biologists.com/dev/article-lookup/doi/10.1242/dev.205258#supplementary-data

Table S3. List of small-spotted catshark genes used for the CellChat analysis. List of 13169 small-spotted catshark orthologs to mouse genes used for the CellChat analysis in Fig. 3F and S6. Details of the BLASTp results are also provided.

Available for download at
https://journals.biologists.com/dev/article-lookup/doi/10.1242/dev.205258#supplementary-data

Table S4. Summary of HCR probe sets used in this study. All HCR probe sets were designed to target transcripts present in the following genome assemblies: small-spotted catshark (*Scyliorhinus canicula*) sScyCan1.1 (GCA_902713615.1), chicken (*Gallus gallus*) bGalGal1.mat.broiler.GRCg7b (GCF_016699485.2), mouse (*Mus musculus*) GRCm39 (GCF_000001635.27).

Gene	Species	Accession number	Obtained from
ADGRD2	Small-spotted catshark	XM_038781796.1	HCR 3.0 probe maker + IDT
ALX1	Small-spotted catshark	XM_038781438.1	HCR 3.0 probe maker + IDT
COL1A1 (LOC119953851)	Small-spotted catshark	XP_038634348.1	Molecular Instruments
CYP26B1 (LOC119963822)	Small-spotted catshark	XM_038793129.1	HCR 3.0 probe maker + IDT
DLX2 (LOC119961904)	Small-spotted catshark	XM_038789414.1	HCR 3.0 probe maker + IDT
EDNRA	Small-spotted catshark	XM_038791956.1	HCR 3.0 probe maker + IDT
EDNRB (LOC119977441)	Small-spotted catshark	XM_038818350.1	HCR 3.0 probe maker + IDT
EN1	Small-spotted catshark	XM_038789834.1	HCR 3.0 probe maker + IDT
FLI1	Small-spotted catshark	XM_038778748.1	HCR 3.0 probe maker + IDT
GAD1 (LOC119961926)	Small-spotted catshark	XM_038789481.1	HCR 3.0 probe maker + IDT
HESX1	Small-spotted catshark	XM_038812822.1	HCR 3.0 probe maker + IDT
KDR	Small-spotted catshark	XM_038791165.1	HCR 3.0 probe maker + IDT
KRT1 (LOC119954220)	Small-spotted catshark	XM_038779258.1	HCR 3.0 probe maker + IDT
LHX2 (LOC119955467)	Small-spotted catshark	XM_038781670.1	HCR 3.0 probe maker + IDT
NEUROD1	Small-spotted catshark	XP_038645181.1	Molecular Instruments
PAX1	Small-spotted catshark	XM_038806068.1	HCR 3.0 probe maker + IDT
PAX7 (LOC119951058)	Small-spotted catshark	XM_038774094.1	HCR 3.0 probe maker + IDT
PHOX2B	Small-spotted catshark	XM_038793037.1	HCR 3.0 probe maker + IDT
PITX3	Small-spotted catshark	XM_038783023.1	HCR 3.0 probe maker + IDT
POSTN	Small-spotted catshark	XM_038817811.1	HCR 3.0 probe maker + IDT
POSTN	Chicken	XM_015277591.4	HCR 3.0 probe maker + IDT
Postn	Mouse	NM_001368678.1	HCR 3.0 probe maker + IDT
SHH	Small-spotted catshark	XM_038798390.1	Molecular Instruments
TBXTB	Small-spotted catshark	XM_038808682.1	HCR 3.0 probe maker + IDT
TWIST1 (LOC119966161)	Small-spotted catshark	XM_038797462.1	HCR 3.0 probe maker + IDT
WNT1 (LOC119958104)	Small-spotted catshark	XM_038786333.1	HCR 3.0 probe maker + IDT
WNT8B	Small-spotted catshark	XM_038783215.1	HCR 3.0 probe maker + IDT
XBP1	Small-spotted catshark	XM_038791411.1	HCR 3.0 probe maker + IDT

Development • Supplementary information

Table S5. Small-spotted catshark gene symbols corresponding to LOC annotations. List of main gene symbols used throughout the scRNA-seq analyses and their corresponding LOC gene annotation from the small-spotted catshark genome sScyCan1.1 (GCA_902713615.1).

Small-spotted catshark LOC gene annotations	Corresponding gene symbols
LOC119958104	*WNT1*
LOC119966161	*TWIST1*
LOC119951058	*PAX7*
LOC119972756	*CCDC102*
LOC119954114	*OPCML*
LOC119961904	*DLX2*
LOC119954093	*TWIST2L*
LOC119967228	*TFAP2B*
LOC119958182	*ERBB4*
LOC119977441	*EDNRB*
LOC119955946	*BARHL1*
LOC119955467	*LHX2*
LOC119957896	*MEIS2*
LOC119963822	*CYP26B1*
LOC119953851	*COL1A1*
LOC119955304	*PODN*
LOC119954220	*KRT1*
LOC119954208	*DLX6L*
LOC119969485	*SNAI1*
LOC119961926	*GAD1*
LOC119974865	*PPIH*
LOC119951944	*SERPINH1*
LOC119974510	*BNC2L*
LOC119965489	*PCDH10*
LOC119977334	*COL4A1*
LOC119964477	*MKNK1*
LOC119970178	*EIF6*
LOC119951038	*2410004B18Rik*
LOC119970372	*IDNK*
LOC119971135	*HNF4AL*
LOC119963518	*UNC5C*
LOC119962784	*ISL1*
LOC119969645	*EYA2*
LOC119951979	*PLP1*
LOC119965024	*LCP2*
LOC119963756	*NEUROG2*
LOC119952095	*SIX1*
LOC119969562	*NKAIN4*
LOC119970772	*EGFLAM*

Supplementary References

Briggs, J. A., Weinreb, C., Wagner, D. E., Megason, S., Peshkin, L., Kirschner, M. W. and Klein, A. M. (2018). The dynamics of gene expression in vertebrate embryogenesis at single-cell resolution. *Science* **360**, eaar5780.

Ibarra-Soria, X., Jawaid, W., Pijuan-Sala, B., Ladopoulos, V., Scialdone, A., Jörg, D. J., Tyser, R. C. V., Calero-Nieto, F. J., Mulas, C., Nichols, J., et al. (2018). Defining murine organogenesis at single-cell resolution reveals a role for the leukotriene pathway in regulating blood progenitor formation. *Nat Cell Biol* **20**, 127–134.

Lange, M., Granados, A., VijayKumar, S., Bragantini, J., Ancheta, S., Kim, Y.-J., Santhosh, S., Borja, M., Kobayashi, H., McGeever, E., et al. (2024). A multimodal zebrafish developmental atlas reveals the state-transition dynamics of late-vertebrate pluripotent axial progenitors. *Cell* **187**, 6742-6759.e17.

Oulion, S., Borday-Birraux, V., Debiais-Thibaud, M., Mazan, S., Laurenti, P. and Casane, D. (2011). Evolution of repeated structures along the body axis of jawed vertebrates, insights from the Scyliorhinus canicula Hox code. *Evol Dev* **13**, 247–259.

Pijuan-Sala, B., Griffiths, J. A., Guibentif, C., Hiscock, T. W., Jawaid, W., Calero-Nieto, F. J., Mulas, C., Ibarra-Soria, X., Tyser, R. C. V., Ho, D. L. L., et al. (2019). A single-cell molecular map of mouse gastrulation and early organogenesis. *Nature* **566**, 490–495.

Qiu, C., Martin, B. K., Welsh, I. C., Daza, R. M., Le, T.-M., Huang, X., Nichols, E. K., Taylor, M. L., Fulton, O., O'Day, D. R., et al. (2024). A single-cell time-lapse of mouse prenatal development from gastrula to birth. *Nature* **626**, 1084–1093.

Rito, T., Libby, A. R. G., Demuth, M., Domart, M.-C., Cornwall-Scoones, J. and Briscoe, J. (2025). Timely TGFβ signalling inhibition induces notochord. *Nature* **637**, 673–682.

Soldatov, R., Kaucka, M., Kastriti, M. E., Petersen, J., Chontorotzea, T., Englmaier, L., Akkuratova, N., Yang, Y., Häring, M., Dyachuk, V., et al. (2019). Spatiotemporal structure of cell fate decisions in murine neural crest. *Science* **364**, eaas9536.

Tirosh, I., Izar, B., Prakadan, S. M., Wadsworth, M. H., Treacy, D., Trombetta, J. J., Rotem, A., Rodman, C., Lian, C., Murphy, G., et al. (2016). Dissecting the multicellular ecosystem of metastatic melanoma by single-cell RNA-seq. *Science* **352**, 189–196.

Wagner, D. E., Weinreb, C., Collins, Z. M., Briggs, J. A., Megason, S. G. and Klein, A. M. (2018). Single-cell mapping of gene expression landscapes and lineage in the zebrafish embryo. *Science* **360**, 981–987.
